# Receptor-Interacting Protein Kinase 3 Suppresses Mitophagy Activation *via* the Yes-Associated Protein/Transcription Factor EB Pathways in Septic Cardiomyopathy

**DOI:** 10.3389/fcvm.2022.856041

**Published:** 2022-03-24

**Authors:** Pingjun Zhu, Yangxiaocao Chen, Junyan Wang, Geng Lin, Runsheng Wang, Yifan Que, Jin Zhou, Guogang Xu, Jiang Luo, Yingzhen Du

**Affiliations:** ^1^Department of Respiratory and Critical Care Medicine, The Second Medical Center and National Clinical Research Center for Geriatric Diseases, Chinese PLA General Hospital, Beijing, China; ^2^Medical Supplies Center, Chinese PLA General Hospital, Beijing, China; ^3^School of Pharmaceutical Sciences, Guangzhou University of Chinese Medicine, Guangzhou, China; ^4^The Second Medical Center and National Clinical Research Center for Geriatric Diseases, Chinese PLA General Hospital, Beijing, China; ^5^Chinese PLA General Hospital, Medical School of Chinese PLA, Beijing, China; ^6^The Eighth Medical Center, Chinese PLA General Hospital, Beijing, China; ^7^Department of Disease Control and Prevention, The Second Medical Center and National Clinical Research Center for Geriatric Diseases, Chinese PLA General Hospital, Beijing, China

**Keywords:** RIPK3, YAP, TFEB, mitophagy, septic cardiomyopathy

## Abstract

Mitophagy, known as the main mechanism of mitochondrial quality control, determines the pathophysiology of septic cardiomyopathy, although the precise regulatory mechanisms remain elusive. Data from the present study suggested that receptor-interacting protein kinase 3 (RIPK3) expression could be enhanced in response to lipopolysaccharide (LPS) challenge. Upregulated RIPK3 expression was accompanied by severe cardiac injury and cardiac dysfunction. Further examination revealed that elevated RIPK3 expression subsequently inhibited the Yes-associated protein (YAP) pathway, which was accompanied by reduced transcription factor EB (TFEB) expression. Inhibition of TFEB would reduce mitophagy, which ultimately induced cardiomyocyte death under LPS challenge. In contrast, loss of RIPK3 induced the YAP/TFEB/mitophagy pathway alleviated the sensitivity of cardiomyocytes to LPS-induced cytotoxicity. Collectively, the RIPK3/YAP/TFEB axis was confirmed to be responsible for the pathogenesis of septic cardiomyopathy by inhibiting mitophagy. These findings have potential significance for the progression of new approaches to the treatment of septic cardiomyopathy.

## Introduction

In general, life-threatening organ dysfunction after infections can be attributed to sepsis secondary to severe systemic inflammation (1–3). Septic cardiomyopathy following sepsis is featured with left ventricular diastolic dysfunction and impaired left ventricular ejection fraction (LVEF), which results in substantially decreased cardiac output and adversely affects blood perfusion of vital organs (4). The prevalence of septic cardiomyopathy has been reported to vary from 40 to 70% (5). Accordingly, septic cardiomyopathy is the primary cause of death among patients with sepsis. Several mechanisms, including inflammatory response, calcium overload, ER stress, oxidative stress, opened mitochondrial permeability transition pore (mPTP) and cell necrosis are the pathogenic factor in septic cardiomyopathy (6, 7). However, the exact molecular features of sepsis-mediated myocardial depression remain unclear. Recent studies have shown the involvement of mitochondrial dysfunction in septic cardiomyopathy, and it acts a pivotal part in cardiac dysfunction and myocardial injury (8, 9). Mitochondrial dysfunction induced by sepsis causes insufficient ATP production, imbalances in cellular oxidative stress, cytokine release, activation of inflammatory response(s), and myocardial death (7). Nevertheless, the upstream mechanism leading to mitochondrial damage in septic cardiomyopathy remains unclear.

Mitophagy is the process that specifically removes damaged mitochondria and alleviates mitochondrial injury *via* the lysosomal pathway (10, 11). The activation of mitophagy also mitigates mitochondrial dysfunction, as indicated by decreased levels of mitochondrial reactive oxygen species (ROS), mitochondrial calcium overload, mitochondrial DNA injury, and mitochondrial apoptosis (12, 13). Mitophagy has been found to be preventive in numerous mankind diseases, including myocardial ischemia (14), acute kidney failure (15), cancer (16), and diabetes (17). Furthermore, our recent study revealed abnormal mitophagy in inflammation-mediated myocardial injury (18). However, the molecular mechanism(s) by which sepsis decreases mitophagy in cardiomyocytes is unclear.

Receptor-interacting protein kinase 3 (RIPK3) is an important factor regulating inflammation-related mitochondrial injury in septic cardiomyopathy (19, 20). Our previous studies also confirmed that RIPK3 deficiency can inhibit mitochondrial damage and myocardial injury by activating mitophagy in cardiac ischemia reperfusion injury and cardiac remodeling after myocardial infarction (14, 21). Thus, we hypothesized that RIPK3 is also involved in the abolition of mitophagy in septic cardiomyopathy. Whereas, the detailed mechanism of RIPK3-mediated mitophagy in septic cardiomyopathy remains unclear.

Transcription factor EB (TFEB) is a basic helix-loop-helix-leucine-zipper (bHLH-Zip) transcription factor, belonging to the microphthalmia family (MiT family). TFEB is well-established as a key regulator of lysosome biogenesis and promotes lysosomal fusion with autophagosomes (22). There is also evidence that TFEB gets involved in the regulation of mitophagy in inflammatory-related diseases. In lipopolysaccharide (LPS)-mediated acute lung injury (ALI), the activation of mitophagy is dependent on the increased expression of TFEB (23). In hepatocytes, TFEB ameliorates liver injury by activating parkin-mediated mitophagy in response to LPS challenge (24). Moreover, RIPK3 contributes to tubular injury and renal insufficiency in sepsis-mediated acute kidney injury (AKI) by inhibiting TEFB expression (25). Therefore, we hypothesized that mitophagy could be regulated by RIPK3 *via* the TEFB pathway in the pathogenesis of septic cardiomyopathy.

## Methods

### Animals and Treatments

The experimental schemes were authorized by the Animal Care and Use Committee of Chinese PLA General Hospital (Beijing, China). Wild-type C57BL/6 mice and C57BL/6 mice with Ripk3^−/−^ (male, 20–22 g, 10–12 weeks old) were obtained from GemPharmatech (21). These mice had abundant food and water supply. Basic environment was settled as controlled temperature (21 ± 2°C) and regular circadian rhythm (12/12 h light/dark cycles). Mice (*n* = 6 per group) were intraperitoneally injected with LPS (6 mg/kg for 48 h) to establish septic cardiomyopathy according to a method described in a previous study (8). After treatment, blood samples were collected to measure the myocardial injury markers, such as creatine kinase-MB (CK-MB), troponin T, and lactate dehydrogenase (LDH) (8).

### Echocardiography and Evaluation of Cardiomyocyte Shortening/Re-lengthening

The heart performance was measured by Echocardiography in all mice 48 h after LPS injection (14.0 MHz transducer, Sequoia C512; Acuson, Siemens, Erlangen, Germany). The cardiomyocytes mechanical properties were assessed with SoftEdge Myocam system (IonOptix, Milton, MA, USA). The changes of cardiomyocyte length were monitored using SoftEdge software (14) and evaluated through the indices as follows: peak shortening (PS), time-to-PS (TPS), time-to-90% re-lengthening (TR90), maximal velocity of shortening (+dL/dt), and re-lengthening (-dL/dt).

### Cell Culture

Cardiomyocytes were separated from Ripk3^−/−^ and wild-type mice *via* the enzyme dissociation (26) and then cultured in high-glucose Dulbecco's Modified Eagle Medium (DMEM; Gibco/Thermo-Fisher Scientific, Waltham, MA, USA). Twenty percentage fetal bovine serum (HyClone Laboratories, Logan UT, USA) was supplied and external condition was settled as 37°C with an atmosphere of 5% CO2 and 95% air. Cardiomyocyte damage was induced by treating cardiomyocytes with LPS (5 μg/ml) in DMEM for 48 h (8).

### ELISA

The concentrations of inflammatory cytokines, including tumor-necrosis factor-alpha (TNF-α), interleukin (IL)-6, IL-10, monocyte chemoattractant protein-1 (MCP1), and cardiac injury markers, including creatine kinase, LDH, and troponin T, were determined with ELISA kits in accordance with manufacturer's protocols.

### Western Blotting

Electrophoresis with a 10% sodium-dodecyl polyacrylamide gel was used to isolate equal amounts (20–35 μg) of total protein, which were subsequently transferred onto an Immobilon-P PVDF membrane. Given the better protein separation efficiency of a gradient gel, the intention was to cut the membrane into small pieces to blot 2–3 proteins. Briefly, membranes were pre-treated with reagents from a protein stain kit for PVDF membranes (Pierce Biotechnologies, Waltham, MA, USA) and were split into several pieces according to the estimated molecular sizes with Spectra Multicolor Broad Range Protein Ladder (cat. no., 26623, Thermo-Fisher Scientific, USA) as the reference. The PVDF membranes were combined with 3% bovine serum albumin (BSA; prepared in 1 × TBST, pH 7.4, same as below) for 60 min at 25°C, and were incubated with the first antibodies in 3% BSA/TBST at 4°C overnight. Next day, the membranes were rinsed 3 times with 1 × TBST for 5 min and then incubated with species-relevant horseradish peroxidase-linked secondary antibodies (diluted 1:500–1:1,000) for 1 h at 20–25°C. The membranes were rinsed with TBST prior to incubation in a commercially available Western blotting detection reagent (ECL Prime Western Blotting Detection Reagent, Amersham cat. No., RPN2232, GE Healthcare, Madison, WI, USA) or Clarity Max Western ECL Substrate (cat. no., 1705062, Bio-Rad Laboratories, Hercules, CA, USA). Target bands were clarified using an ImageQuant LAS 400 system (GE Healthcare, USA) and band luminosity was quantified using the inbuilt ImageQuant TL software version 7.0 (GE Healthcare, USA) and standardized to GADPH.

### Immunofluorescence Assays

After treatment, the samples were washed in phosphate-buffered saline (PBS) for 3 times and fixed using 4% paraformaldehyde at 25°C for 30 min. Then, the samples were incubated using Ripk3 (1:1,000 dilution, cat. no., 95702, Cell Signaling Technology, Danvers, MA, USA) and cardiac troponin T (1:1000, ab50576, Abcam, Cambridge, United Kingdom) antibodies at 4°C for 12 h. Subsequently, the samples were rinsed with PBS 3 times, followed by staining with fluorescent secondary antibody for 30 min at 37°C. DAPI was applied for nuclear staining. Fluorescence microscope (Olympus Corporation, Tokyo, Japan) was used for image taking.

### Quantitative Real-Time Polymerase Chain Reaction

qRT-PCR was performed according to a standard protocol. The primers below were used for PCR: LAMP1, forward, 5′-ACTGGTAACAACGGAACCTG−3′, reverse, 5′-ACACATTGGGGTTAGGAACA−3′; LAMP2, forward, 5′-CTAGGAGCCGTTCAGTCCAA−3′, reverse: 5′-CTTGCAGGTGAATACCCCAA−3′; GADPH, forward: 5′-TGGAGTCTACTGGCGTCTT−3′, reverse: 5′-TGTCATATTTCTCGTGGTTCA−3′. Concentrations of mRNA were measured using qRT-PCR for each RNA in triplicate, with GADPH as the endogenous control.

### RNA Interference

TFEB and YAP were knocked down in RIPK3^−/−^ cells using small interfering RNAs (siRNA). We purchased siRNAs and their non-targeting sequences (negative controls) from GenePharma Co., Ltd., Shanghai, China.

### Measurement of Mitochondrial Membrane Potential, mPTP Opening, and ATP Content

Changes in *mitochondrial membrane potential* (ΔΨm) were evaluated with the JC-1 Kit (Beyotime, China). mPTP opening was evaluated according to the rapid dissipation of tetramethylrhodamine ethyl ester (TMRE) fluorescence, in accordance with a method described in a previous study by the authors (27). The ATP generation was assessed with a commercially available ATP bioluminescence assay kit (Beyotime, China) as described previously (28).

### ROS, MTT, and Caspase-3 Activity Assays

ROS were measured using dihydroethidium (Invitrogen, San Diego, CA, USA) staining and assessed with a microscope according to our previous study (21). Cellular viability was evaluated by the 3-(4,5-dimethylthiazol-2-yl)-2,5-diphenyltetrazolium bromide (MTT) assay (29). A commercially available caspase 3 colorimetric assay kit (cat. no., APT165, Millipore/Sigma, Burlington, MA, USA) was also used for assessment.

### Data Analysis and Statistics

All variables are presented as mean ± standard error of mean (SEM). One- or two-way ANOVA and Student-Newman-Keuls *post-hoc* tests or *t*-tests were used for statistical analysis. *P*-value < 0.05 was considered statistically significant.

## Results

### RIPK3 Deficiency Inhibited LPS-Mediated Myocardial Injury

**Figure 1:**
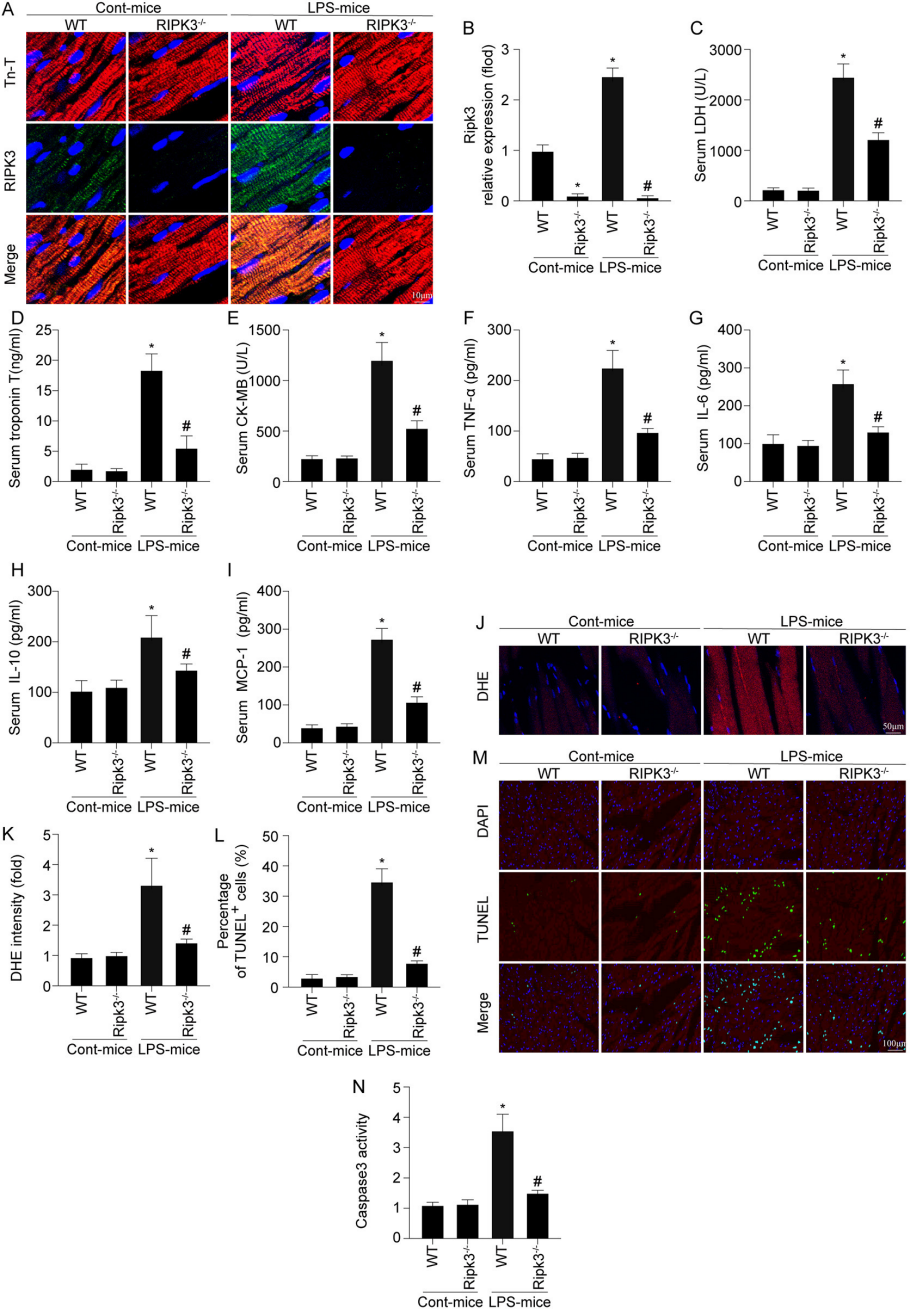
RIPK3 deletion prevents LPS-mediated cardiac injury (*n* = 6/group). **(A,B)** LPS was injected into WT and Ripk3^−/−^ mice to establish the septic cardiomyopathy model. Then, RIPK3 expression was measures *via* Immunofluorescence. **(C–E)** Subsequently, blood was isolated and ELISAs were used to detect the alterations of the cardiac damage markers LDH, CK-MB and Tn-T. **(F–I)** the concentrations of TNFα, IL-6, IL-10 and MCP-1 in blood were measured *via* ELISAs. **(J,K)** The ROS levels in cardiac tissue was detected through DHE staining. TNNEL assay **(L,M)** and the caspase-3 activity **(N)** were used to detect cardiomyocyte death. *p < 0.05 vs. WT group. ^#^*p* < 0.05 vs. WT + LPS group.

First, alterations in RIPK3 in the heart was measured *via* immunofluorescence after LPS treatment. As shown in Figures 1A,B, LPS treatment significantly enhanced RIPK3 expression compared to the control group. RIPK3^−/−^ mice were used to further investigate the role of RIPK3 in septic cardiomyopathy subsequently. LPS treatment notably enhanced the serum levels of myocardial injury markers comparing to the control group (Figures 1C–E). Interestingly, RIPK3 deletion notably decreased the concentrations of myocardial injury markers. An enhanced inflammatory response and oxidative stress have been shown to be involved in the pathophysiology of sepsis-induced cardiomyopathy. As shown in Figures 1F–K, LPS-mediated sepsis significantly increased inflammatory cytokines than the control group, including TNF-α, IL-6, IL-10, MCP1, as well as ROS production, in the mouse heart. However, compared to the LPS group, knockout of RIPK3 significantly reduced the levels of inflammatory factors and ROS production. Cardiomyocyte death is a well-established marker of myocardial injury caused by sepsis; thus, the TUNEL assay and caspase-3 activity were used to detect cardiomyocyte death. As shown in Figures 1L–N, the LPS-treated group had higher percentage of TUNEL-positive cells and caspase-3 activity in heart tissue than that in the control group. However, the loss of RIPK3 reduces LPS-induced cardiomyocyte death. Based on the above findings, sepsis-induced myocardial injury was associated with upregulated expression of RIPK3.

### Loss of RIPK3 Preserved Cardiac Function Under LPS Challenge

**Figure 2:**
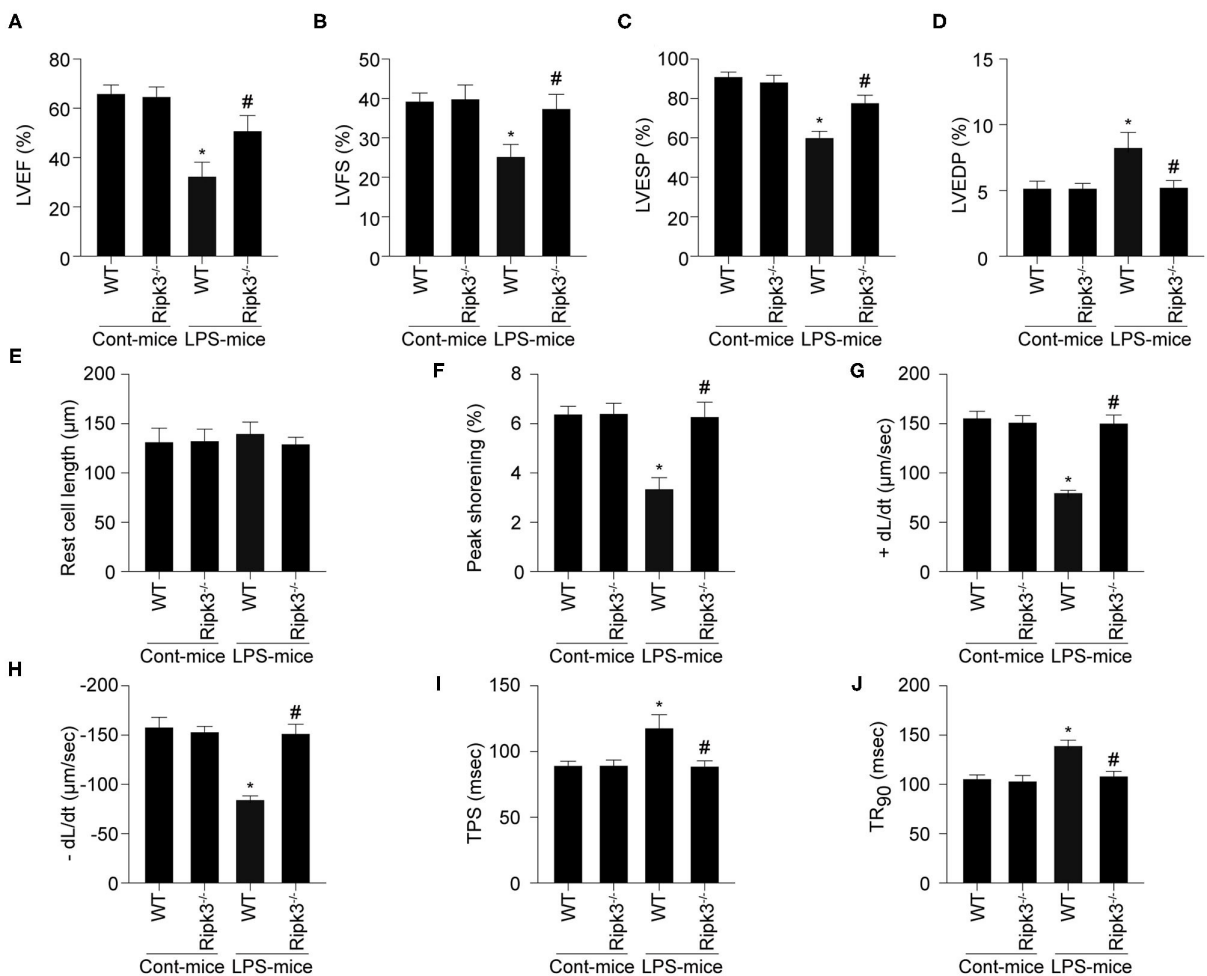
LPS-induced cardiac dysfunction could be improved by RIPK3 deletion (*n* = 6/group). **(A–D)** Cardiac function was measured *via* echocardiography. **(E–J)** Cardiomyocyte mechanical parameters were monitored using a VEVO 2,100 high-resolution imaging system. Cardiomyocytes were isolated from LPS-treated mice and melatonin-treated mice. Then, the resting cell length, peak shortening, time-to-90% re-lengthening (TR90), time-to- shortening (TPS), the maximal velocity of shortening (+dL/dt) and the maximal velocity of re-lengthening (-dL/dt) were determined. *p < 0.05 vs. WT group. ^#^*p* < 0.05 vs. WT + LPS group.

Cardiac function was also measured using echocardiography. Comparing with the control group, LPS treatment significantly reduced LVEF, LV fractional shortening (LVFS), and LV end-systolic pressure (LVESP), and increased LV end-diastolic pressure (LVEDP) (Figures 2A–D). Interestingly, RIPK3 deletion largely reversed LPS-induced alterations in cardiac function parameters. Subsequently, changes in cardiomyocyte contraction properties were characterized by acute isolation of cardiomyocytes from wild-type and RIPK3^−/−^ mice according to our previous work. As shown in Figure 2E, LPS treatment and genetic ablation of RIPK3 had no effect on cardiomyocyte length. However, compared to the control group, LPS impaired the contractile and diastolic functions of cardiomyocytes, accordance with the remarkably reduced PS, ± dL/dt, TR90, and TPS (Figures 2F–J). In contrast, LPS-induced myocardial dysfunction was shown to be decreased significantly or reversed by the loss of RIPK3. Collectively, these results confirmed that knockout of RIPK3 preserves cardiac function in patients with septic cardiomyopathy.

### LPS-Activated RIPK3 Promoted Mitochondrial Injury *via* Inhibiting Mitophagy

**Figure 3:**
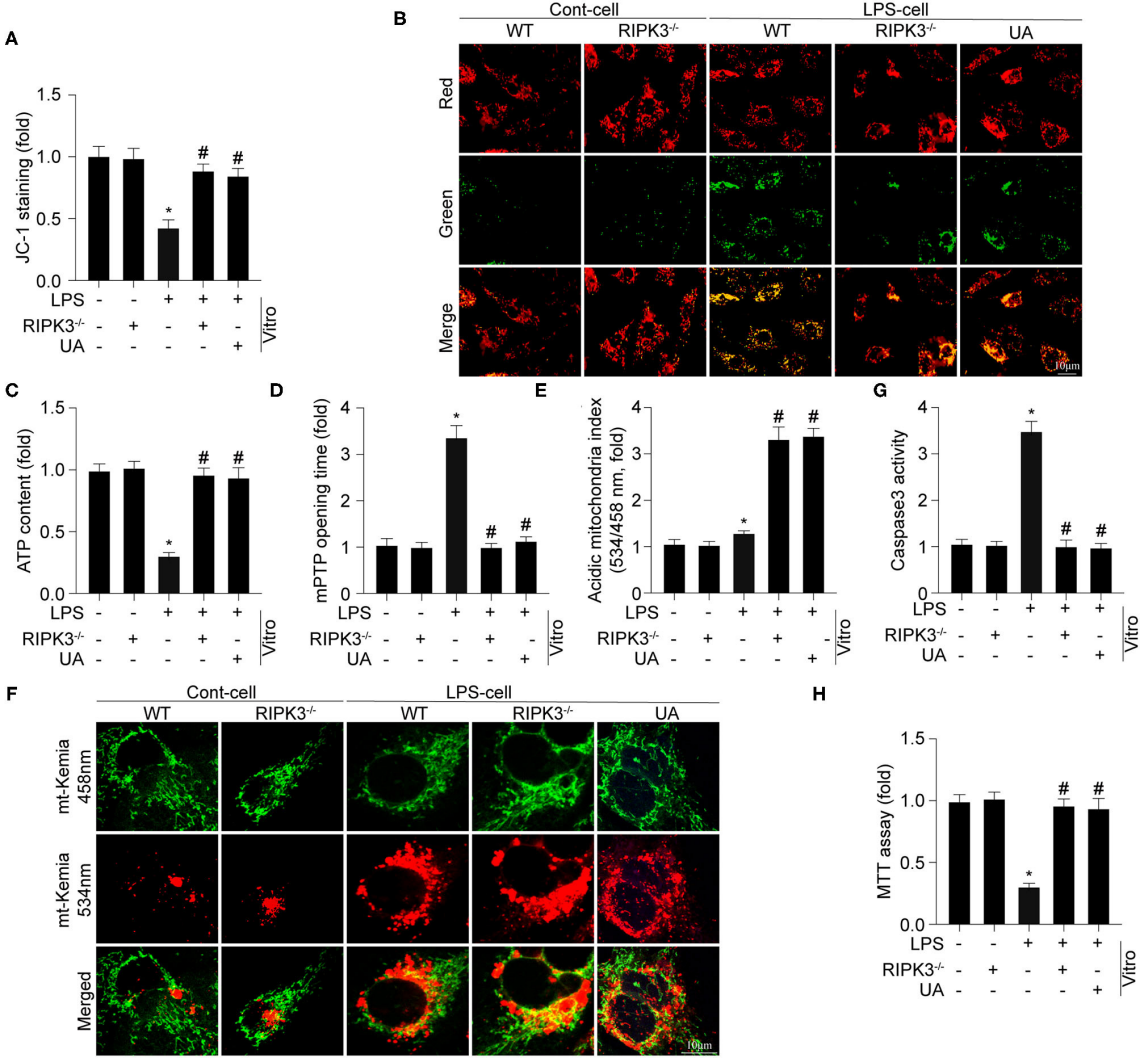
Genetic ablation of RIPK3 sustains mitochondrial injury *via* enhancing mitophagy (*n* = 6/group). **(A,B)** Cardiomyocytes were isolated from WT and RIPK3 mice and then treated with LPS (5 μg/ml) to to induce sepsis-related cardiomyocyte damage. urolithin A (UA), an inducer of mitophagy, was used to culture cardiomyocytes. The mitochondrial membrane potential (ΔΨm) was measured by JC-1 assay. **(C)** ATP generation was measured to reflect mitochondrial energy metabolism. **(D)** Arbitrary mPTP opening time was determined as the time when the TMRE fluorescence intensity decreased by half between the initial and residual fluorescence intensity. **(E,F)** Mitophagy activity was observed using the mt-Keima assay. A yellow signal highlights increased mitophagic flux in cardiomyocytes. Caspase3 activity **(G)** and MTT assay **(H)** were used to measure the alteration of cardiomyocytes viability. *p < 0.05 vs. WT group. ^#^*p* < 0.05 vs. WT + LPS group.

Previous studies have reported that mitochondrial damage is a hallmark feature of sepsis-mediated myocardial injury. First, mitochondrial membrane potential was determined using an *in vitro* JC-1 assay (Figures 3A,B). LPS treatment significantly reduced mitochondrial membrane potential in cardiomyocytes when comparing to the control group. Knockout of RIPK3 stabilized mitochondrial membrane potential to near-normal levels in LPS-treated cells. Furthermore, cellular ATP generation was decreased following LPS treatment, which was reversed by RIPK3 loss (Figure 3C). Compared with the control group, the mPTP opening time in LPS-treated cells was found to be significantly longer (Figure 3D). However, loss of RIPK3 significantly decreased mPTP opening time following LPS treatment. A previous study by the authors demonstrated the cardioprotective role of mitophagy in LPS-mediated mitochondrial injury and septic cardiomyopathy. A focus on mitophagy was used to explore the underlying mechanism by which RIPK3 promotes mitochondrial injury. Urolithin A (UA), mostly used as an inducer of mitophagy, was applied in cardiomyocytes under LPS challenge. Mitophagy activity of cardiomyocytes was assessed using the Mt-Keima assay. Compared to the control group, mitophagy was moderately and intensely activated by LPS and UA, separately (Figures 3C,E,F). However, RIPK3 knockout largely promoted activation of mitophagy, which was consistent with the application of UA in response to LPS. Furthermore, UA also inhibited LPS-induced mitochondrial injury as indicated by enhanced mitochondrial membrane potential, increased ATP generation, and decreased mPTP opening time, which was like the loss of RIPK3. To gain more evidence, cardiomyocyte death was also evaluated using caspase 3 activity (Figure 3G) and MTT assay (Figure 3H). According to current study, LPS induction notably reduced cardiomyocyte viability and increased cardiomyocyte death, which could be reversed by knockout of RIPK3. Thus, these results confirmed that the mitochondrial injury mediated by RIPK3 during septic cardiomyopathy could be inhibited and alleviated by the activated mitophagy.

### Knockout of RIPK3 Modulated Mitophagy by the Up-Regulation of TFEB

**Figure 4:**
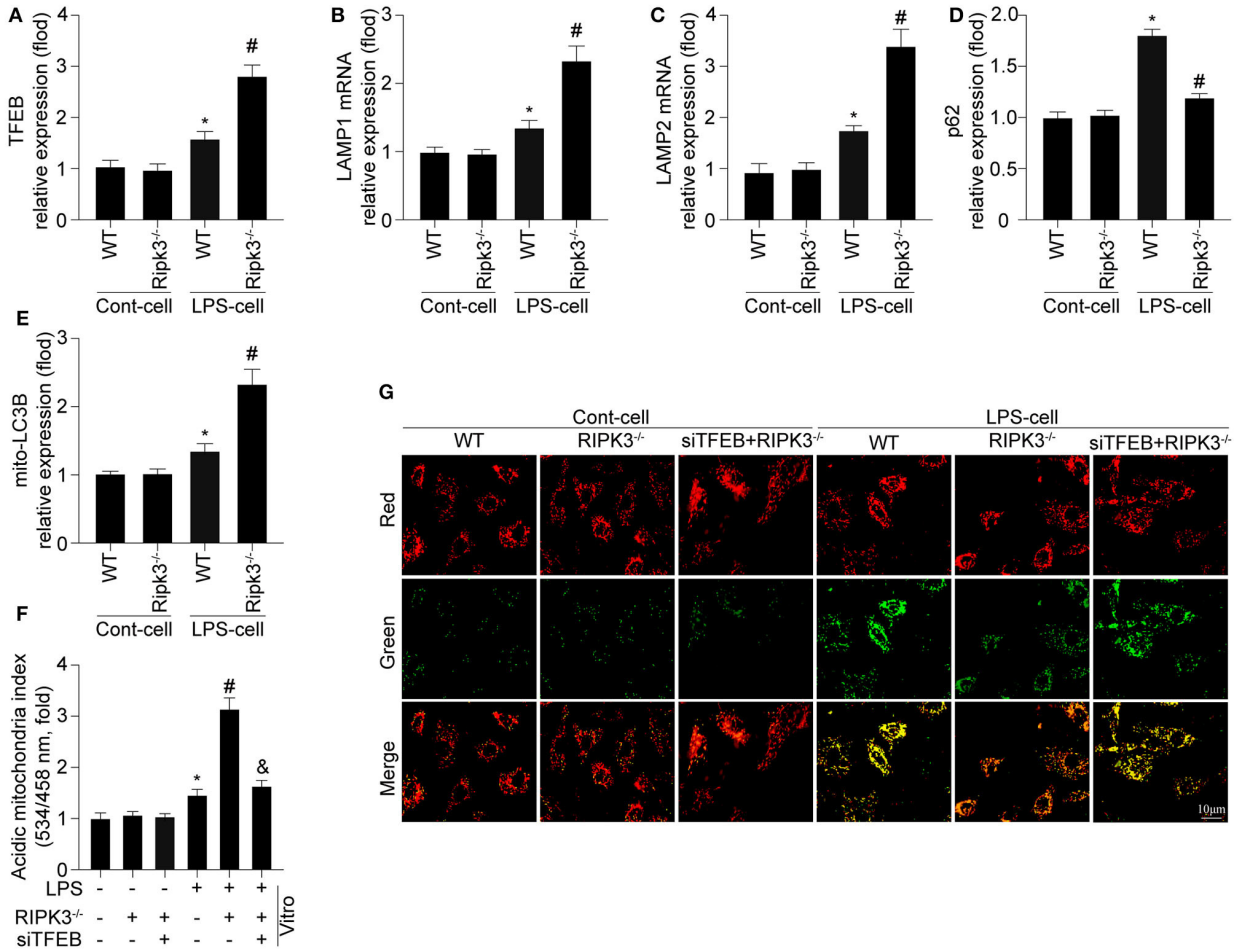
Mitophagy is modified by RIPK3 in a manner dependent on TFEB expression (*n* = 6/group). **(A)** Cardiomyocytes were isolated from WT and Ripk3^−/−^ mice and then treated with LPS (5 μg/ml) to induce sepsis-related cardiomyocyte damage. The expression of TFEB was measured *via* western blot. **(B,C)** The alteration of LAMP1 and LAMP2 mRNA expression were measured *via* qRT-PCR. **(D,E)** mito-LC3B and p62 were measured *via* western blot. **(F)** siRNA against TFEB (si-TFEB) was transfected into RIPK3^−/−^ cells to reduced TFEB expression under LPS challenge. ΔΨm was measured by JC-1 assay. **(G)** Mitophagy activity was observed using the mt-Keima assay. *p < 0.05 vs. WT group. ^#^*p* < 0.05 versus WT + LPS group. ^&^*p* < 0.05 vs LPS + RIPK3^−/−^ group.

According to previous studies, the disruption of mitophagy is associated with lysosomal dysfunction (24, 30). Given the pivotal role of TFEB in myocardial lysosomal biogenesis, we detected the expression of TFEB and mRNA of key lysosomal proteins (LAMP1 and LAMP2). As shown in Figures 4A–C, deletion of RIPK3 enabled a more extensive expression of TFEB at the protein level, and expression of LAMP1 and LAMP2 at the mRNA level than those in the LPS group, confirming the negative regulatory effect of RIPK3 on TFEB and subsequent lysosomal biogenesis. Furthermore, the upregulated TFEB expression by RIPK3 knockout was consistent with activation of mitophagy as indicated by enhanced mito-LC3B and reduced p62 expression in respond to LPS treatment (Figures 4D,E). To further explore whether TFEB is required for RIPK3 induced mitophagy inhibition, siRNA against TFEB (si-TFEB) was transfected into RIPK3^−/−^ cells to reduce TFEB expression. As shown in Figures 4F,G, si-TFEB inhibited mitophagy and reduced mitochondrial potential in RIPK3^−/−^ cells. These results confirmed that upregulated RIPK3 reduced mitophagy activation by inhibiting TFEB-mediated lysosomal biogenesis.

### Inhibitory Effects of RIPK3 on TFEB Were YAP Dependent

**Figure 5:**
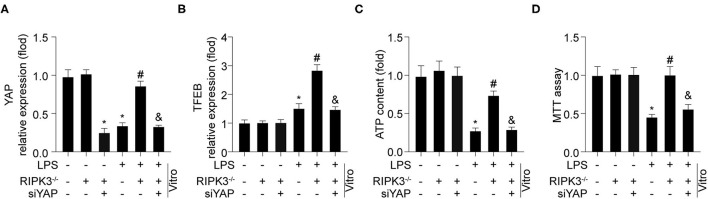
RIPK3 controls TFEB mediated cardiac protection *via* YAP pathway (*n* = 6/group). **(A)** Cardiomyocytes were isolated from WT and RIPK3^−/−^ mice and then treated with LPS (5 μg/ml) to to induce sepsis-related cardiomyocyte damage. siRNA against YAP (si-YAP) was transfected into RIPK3^−/−^ cells to reduced TFEB expression under LPS challenge. YAP **(A)** and TFEB **(B)** expression was detected *via* western blot. **(C)** TP generation was measured by an ATP Bioluminescence Assay Kit. **(D)** MTT assay were used to measure cardiomyocytes viability. *p < 0.05 vs. WT group. ^#^*p* < 0.05 vs. WT + LPS group. ^&^*p* < 0.05 vs. LPS + RIPK3^−/−^ group.

To explore the mechanism by which RIPK3 regulates TFEB, the YAP pathway was explored. As shown in Figure 5A, LPS stimulation reduced YAP expression compared to that in the control group. However, RIPK3 knockout increased YAP expression in response to LPS. To verify whether YAP was required for RIPK3-mediated TFEB inactivation, siRNA against YAP (siYAP) was transfected into RIPK3^−/−^ cells under LPS treatment. As shown in Figure 5B, blockade of the YAP pathway abolished the inhibitory effects of RIPK3 deletion on TFEB. Subsequently, it was investigated whether RIPK3 modulated mitochondrial function and cardiomyocyte viability *via* the YAP/TFEB pathway. The ATP generation analysis revealed that LPS-mediated ATP reduction could be reversed by RIPK3, and this effect was ineffective once the YAP pathway was blocked (Figure 5C). Furthermore, cardiomyocyte viability decreased under LPS treatment but increased to normal levels on RIPK3 deletion. The inhibition of YAP by siRNA in RIPK3^−/−^ cells reduced cardiomyocyte viability (Figure 5D). Collectively, these results demonstrate that RIPK3 controls TFEB-mediated cardiac protection *via* the YAP pathway.

## Discussion

Usually presented as extremely critical organ malfunction, sepsis usually led to dysregulated host response (31). Despite the development of various therapeutic strategies, it remains a leading cause of death around worldwide (8). It is evident that RIPK3 is key in the pathophysiological processes of septic cardiomyopathy (32, 33). However, the mechanism by which RIPK3 mediates septic cardiomyopathy remains unclear. Present study demonstrated that RIPK3 is activated in mice with septic cardiomyopathy. Upregulated RIPK3 contributes to the inflammatory response, oxidative stress, cardiomyocyte death, and cardiac dysfunction. Mechanistically, increased RIPK3 inhibited TFEB and lysosome lysosomal biogenesis *via* the YAP pathway, which suppressed mitophagy activity. These findings highlight the pivotal role of the RIPK3/YAP/TFEB pathway implicated in mitophagy suppression during septic cardiomyocytes, thus providing potential therapeutic targets to early intervention and clinical treatment of septic cardiomyopathy.

During sepsis, inflammation-induced mitochondrial injury determines in the pathogenesis of myocardial dysfunction (8, 9). In this study, our results confirmed that sepsis-induced myocardial mitochondrial dysfunction was characterized by ATP shortage, reduction in mitochondrial membrane potential, mitochondrial ROS outburst, and activation of cardiomyocyte death. The increased mitochondrial injury was due to reduced clearance of damaged mitochondria by mitophagy in the pathological progression of septic cardiomyopathy (34). These findings were consistent with previous reports (18, 35), in which mitophagy reduced cardiac mitochondrial injury *via* inhibiting mitochondrial injury. Thus, our study provides a survival advantage against septic cardiomyopathy.

In our current research, we mainly identified the upstream regulatory mechanism responsible for mitophagy in septic cardiomyopathy. Previous studies have confirmed that lysosome dysfunction-mediated incompletion of mitophagy may contribute to sepsis-induced mitochondrial injury (24, 36). Other studies confirmed that activation of lysosome function alleviates mitochondrial damage by promoting the fusion of mitochondria and lysosomes (37, 38). However, the underlying mechanism of lysosomal dysfunction in septic cardiomyopathy remains vaguely. Herein we confirmed that RIPK3 inhibited mitophagy *via* TFEB-mediated lysosomal dysfunction in response to treatment with LPS. Our previous studies have elaborated that mitophagy is regulated by specific receptors, including Fundc1, Bnip3, and Parkin (19, 21, 39). In IPEC-J2 cells treated with hydrogen peroxide, TEFB inhibited mitophagy *via* the parkin pathway (40). Furthermore, our recent study found that activating Fundc1 associated mitophagy attenuates LPS-induced mitochondrial injury and cardiac damage (18). Further studies are needed to confirm whether TFEB regulates mitophagy *via* the Fundc1 pathway.

RIPK3, a necroptosis-regulating kinase, has been found to be closely associated with lysosomal biogenesis (41, 42). In TNF-treated murine L929 fibroblasts, RIPK3 promoted necroptosis by blocking lysosomal degradation of autophagosomes (43). In septic acute kidney injury, upregulated RIPK3 contributes to lysosomal dysfunction and the accumulation of autophagosomes in renal proximal tubular epithelial cells (25). However, previous studies also reported that the lysosomal function contributed to regulation of RIPK3 (41, 44). In spinal cord injury (SCI), lysosomal damage contributed to accumulation of RIPK3 proteins and followed necroptosis. Herein we demonstrated that the loss of RIPK3 promotes lysosome function and lysosomal degradation of impaired mitochondria, which attenuates mitochondrial injury and cardiac dysfunction. Therefore, the cross-talk between RIPK3 and lysosomal biogenesis might differ depending on the stimulus exposure and disease model.

We next explored how RIPK3 regulates TFEB. Activation of YAP in the heart promotes cardiomyocyte survival in septic cardiomyopathy (45). In this study, the expression of YAP was downregulated in the presence of LPS, indicating that the YAP pathway was inhibited. The YAP pathway has been confirmed to be an important upstream target of TFEB (46, 47). YAP promotes cardiomyopathy through the activation of TFEB in lysosomal storage disorder (46). Furthermore, in doxorubicin-treated H9c2 cells, YAP enhanced parkin-mediated mitophagy and attenuated cell apoptosis (48). Thus, to explore the underlying mechanism by which RIPK3 regulates TFEB, YAP pathway was focused. In this study, we confirmed that RIPK3 reduces TFEB *via* the YAP pathway, hoping to provide therapeutic targets in the intervention of septic cardiomyopathy and further investigation is expected.

## Data Availability Statement

The raw data supporting the conclusions of this article will be made available by the authors, without undue reservation.

## Ethics Statement

The animal study was reviewed and approved by the Animal Care and Use Committee of Chinese PLA General Hospital (Beijing, China).

## Author Contributions

GX, PZ, JL, and YD conceived the project and designed the research. PZ and YD performed the experiments. PZ and YC analyzed the data, discussed the research, and wrote the manuscript. All authors contributed to the article and approved the submitted version.

## Funding

This study was financially supported by Beijing Natural Science Foundation (No. 7222166), National Natural Science Foundation of China (No. 81900254), National key Research and Development Program of China (Nos. 2020YFC2002706 and 2020YFC2008900), and Transformation Project of Chinese PLA General Hospital (No. ZH19027). The funders had no role in the study design, data collection and analysis, decision to publish, or preparation of the manuscript.

## Conflict of Interest

The authors declare that the research was conducted in the absence of any commercial or financial relationships that could be construed as a potential conflict of interest.

## Publisher's Note

All claims expressed in this article are solely those of the authors and do not necessarily represent those of their affiliated organizations, or those of the publisher, the editors and the reviewers. Any product that may be evaluated in this article, or claim that may be made by its manufacturer, is not guaranteed or endorsed by the publisher.
